# Intracellular degradation of functionalized carbon nanotube/iron oxide hybrids is modulated by iron via Nrf2 pathway

**DOI:** 10.1038/srep40997

**Published:** 2017-01-25

**Authors:** Dan Elgrabli, Walid Dachraoui, Hélène de Marmier, Cécilia Ménard-Moyon, Dominique Bégin, Sylvie Bégin-Colin, Alberto Bianco, Damien Alloyeau, Florence Gazeau

**Affiliations:** 1 Laboratoire Matière et Systèmes Complexes, UMR7057 CNRS/Université Paris Diderot, 75013 Paris, France; 2 Laboratoire Matériaux et Phénomènes Quantiques, UMR7057 CNRS/Université Paris Diderot, 75013 Paris, France; 3 CNRS, Institut de Biologie Moléculaire et Cellulaire, Laboratoire d’Immunopathologie et Chimie Thérapeutique, 67000 Strasbourg, France; 4 Institut de Chimie et des Procédés pour L’Energie, l’Environnement et la Santé (ICPEES) UMR 7515, Université de Strasbourg, 25 rue Becquerel, 67087 Strasbourg cedex 2, France; 5 Institut de Physique et de Chimie de Strasbourg (IPCMS) UMR 7504 CNRS-Université de Strasbourg, 23 rue du Loess, BP 34, 67034 Strasbourg cedex 2, France

## Abstract

The *in vivo* fate and biodegradability of carbon nanotubes is still a matter of debate despite tremendous applications. In this paper we describe a molecular pathway by which macrophages degrade functionalized multi-walled carbon nanotubes (CNTs) designed for biomedical applications and containing, or not, iron oxide nanoparticles in their inner cavity. Electron microscopy and Raman spectroscopy show that intracellularly-induced structural damages appear more rapidly for iron-free CNTs in comparison to iron-loaded ones, suggesting a role of iron in the degradation mechanism. By comparing the molecular responses of macrophages derived from THP1 monocytes to both types of CNTs, we highlight a molecular mechanism regulated by Nrf2/Bach1 signaling pathways to induce CNT degradation via NOX_2_ complex activation and O_2_^•−^, H_2_O_2_ and OH^•^ production. CNT exposure activates an oxidative stress-dependent production of iron via Nrf2 nuclear translocation, Ferritin H and Heme oxygenase 1 translation. Conversely, Bach1 was translocated to the nucleus of cells exposed to iron-loaded CNTs to recycle embedded iron. Our results provide new information on the role of oxidative stress, iron metabolism and Nrf2-mediated host defence for regulating CNT fate in macrophages.

The unique properties of carbon nanotubes (CNTs) have allowed the exploration of a plethora of applications in various fields, such as electronics, energy storage and conversion, sensors, automotive and nanomedicine^1^,^2^,^3^,^4^,^5^. Associating CNTs with metal oxide nanoparticles (NPs) is an even more promising approach since the properties of each material can be combined advantageously. While CNTs exhibit outstanding electrical and thermal conductivities, mechanical properties and high specific surface area, magnetic iron oxide nanoparticles have shown great promises for biotechnology, sensing, data storage as well as imaging and therapeutic applications. Nevertheless the long term fate and biodegradability in the body of CNTs and metal oxide/CNT hybrids is still a subject of debate that considerably slows their development and raises serious health issues. A recent study pointed out the presence of multi-walled CNTs in the airways of asthmatic parisian children, identified by transmission electron microscopy into macrophages of broncho-alveolar lavage-fluids^6^. Based on potential asbestos-like pathogenicity, the long term fate of anisotropic carbon materials raises specific concerns related to their architecture^7^. Non-functionalized MWCNTs longer than 20 μm were found to trigger an inflammatory response and result in granuloma formation similar to long asbestos fibres^8^. However, the pathogenic effects of CNTs can be modulated or suppressed by an appropriate functionalization strategy that increases the dispersibility of the material and reduces the aggregation phenomena^9^. Some CNTs have been associated with iron oxide nanoparticles for biomedical applications combining magnetic resonance imaging, hyperthermia therapy, and magnetic manipulation^10^,^11^,^12^,^13^. We recently reported the design of biocompatible functionalized MWCNTs filled with iron oxide NPs, the inner cavity of CNTs acting as a nanoreactor for *in situ* growth of ferrite NPs. These iron oxide/CNT hybrids were efficiently internalized in tumor cells without toxicity, allowing to control CNT uptake and orientation within the cell by magnetic fields and to induce submicron magnetic stirring. In addition to magnetic resonance imaging (MRI) detectability, we also demonstrated that the photothermal ablation of tumor cells could be enhanced by magnetic stimulus, harnessing the hybrid properties of iron oxide loaded-CNTs^11^. Given the high potential of such nanohybrids, it is of utmost importance to decipher the mechanisms of cellular processing and assess their intracellular degradability which may differ from empty iron-free CNTs. Recent studies have shown that oxidized CNTs can be degraded by specific peroxidases, like myeloperoxidase, which is overexpressed in activated neutrophils^14^, and eosinophil peroxidase^15^, relying on their ability to convert H_2_O_2_ into strong oxidants capable of oxidizing CNTs. The enzymatic activity of the peroxidases can be also boosted by modifying CNTs with ligands able to interact with the enzymes and enhance the catalytic activity^16^. Nevertheless the intracellular degradability of CNTs is still debated^17^,^18^. Non-enzymatic degradation medium constituted by phagolysosomal elements associated with H_2_O_2_ was found to be able to induce CNT degradation^19^. Previous studies have shown the capability of CNTs to induce oxidative stress in macrophage cell lines^20^,^21^. These immune cells are well-known as a first line of defence against pathogens and are also the primary responders to different particles that initiate and propagate inflammatory reactions^22^. In the body, CNTs are first engulfed by macrophages as observed in the lungs of rats after intratracheal instillation^23^,^24^, or in the Kupffer cells of liver after intravenous administration^25^. In these types of cells, a slow degradation mechanism occurs in phagosomes demonstrated by structural damages of the carbon structure^23^,^26^. Peroxynitrite-induced CNT degradation was reported to play a significant, but not exclusive, role in the biodegradation process^27^, and in a recent work, we have revealed another important way adopted by macrophages to degrade MWCNTs into intracellular compartments^18^. The internalization of MWCNTs in phagosomes was shown to be associated with the activation of proteins to form an activated NOX_2_ complex on cytosolic and phagosomal membranes. When activated, NOX_2_ complex induced the generation of reactive oxygen species (ROS) and especially superoxide radical O_2_^•−^. Into phagosome, O_2_^•−^ is transformed into H_2_O_2_ by superoxide dismutase (SOD) and H_2_O_2_ is then turned into hydroxyl radical (OH^•^) in the presence of Fe^2+^ and Fe^3+^ (Haber-Weiss reaction). Importantly, OH^•^ was shown to play a crucial role in CNT degradation mechanism because it can attack MWCNT defects and unsaturated carbon bonds on the sidewalls of CNTs to generate carboxylic acids, thus creating holes in the graphitic structure^18^. Importantly *in situ* degradation of CNTs could be directly observed in real time using the new technology of *in situ* TEM in liquid medium. The radiolysis of water induced by the electron beam on CNT suspension generates the formation of ROS that induce the structural degradation of CNTs. This oxidative transformation of CNTs observed *in situ* recapitulates the ROS-induced degradation observed in cells^18^. We observed OH^•^-dependent CNT degradation on both 40–80 nm diameter MWCNTs without iron residue and the same MWCNTs filled with about 6% w/w of iron oxide NPs (Fe@MWCNTs)^11^. In the present study, we aim to go deeper into the intracellular molecular mechanism leading to macrophagic degradation of functionalized MWCNTs filled with iron oxide NPs in a comparative way to iron-free MWCNTs, both being designed for biomedical applications. In particular, we highlight the role of iron and of nuclear factor erythroid 2-related factor 2 (Nrf2) in controlling oxidative damage of Fe@MWCNTs and MWCNTs in macrophages. Nrf2 is a basic leucine zipper transcription factor that has recently emerged as a critical factor of defence against oxidative stress. Nrf2 regulates the expression of antioxidant proteins and drug metabolizing enzymes such as heme oxygenase 1 (Hmox-1). The protective role of Nrf2 in response to toxicants, including metal nanoparticles^28^, or CNTs^29^, has been highlighted recently. Here we investigate for the first time the interplay of CNT-induced oxidative stress, CNT degradation, Nrf2 transcription factor and iron metabolism in macrophages. We reveal that ROS-induced MWCNT degradation can be modulated by exogenous sources of iron through Nrf2 pathway.

## Results

### MWCNT and Fe@MWCNT oxidation and degradation in macrophages

**Figure 1:**
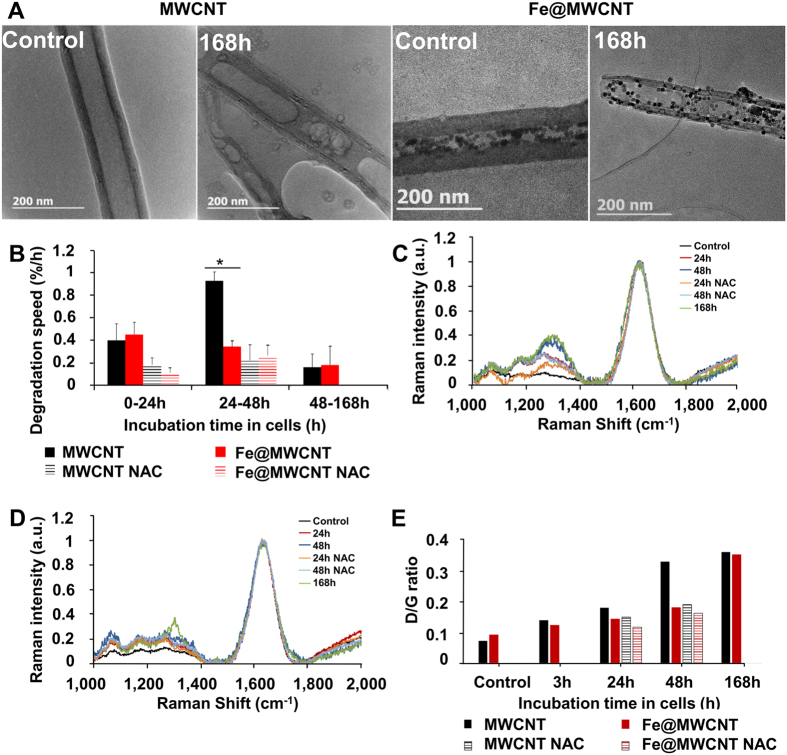
MWCNT and Fe@MWCNT degradation in THP-1 differentiated into macrophages. Cells were exposed to a 1 μg/cm^2^ suspension of MWCNTs or Fe@MWCNTs for 24 h. Non- phagocytosed CNTs were removed and (**A**) TEM observations of extracted MWCNTs or Fe@MWCNTs before (control) and after 168 h aging into cells were performed, showing stimagta of degradation. (**B**) The degradation speed of MWCNTs and Fe@MWCNTs is calculated for different period of aging in cells in the presence or absence of NAC. Raman spectrum of (**C**) MWCNTs and (**D**) Fe@MWCNTs for different times of aging in macrophages in presence or absence of NAC, confirming surface modifications over time. (**E**) D band/G band ratios of Raman spectra. (*) designates a statistically-significant difference between MWCNT and Fe@MWCNT groups (p < 0.05). ($) designates a statistically-significant difference between MWCNT NAC or Fe@MWCNT NAC group and its respective equivalent MWCNT or Fe@MWCNT group without NAC treatment (p < 0.05). Experiments were repeated at least three times with similar observations in each experiment.

THP-1-derived macrophages were exposed to MWCNTs and Fe@MWCNTs added to the culture medium for 24 h. CNTs were extracted from the cells at different time-points after exposure to investigate their structural transformations due to aging in the phagolysosomes of macrophages. Transmission electron microscopy (TEM) revealed that the major aging stigmata on both MWCNTs and Fe@MWCNTs are the creation of holes in the graphitic structure as illustrated in Fig. 1A. The percentage of perforated area defined as perforated area divided by total CNT surface was calculated as a function of the aging time in the cells. First holes were quickly formed, soon after 3 h inside the cells and approximately 2% of the total graphitic surface was degraded in both CNTs at this time point. Holes became bigger over time, but more rapidly for MWCNTs than for Fe@MWCNTs. The surface of holes reached 19.0 ± 5.5% at 48 h and 40.5 ± 7.0% at 168 h for Fe@MWCNTs versus 31.9 ± 4.9% and 51.1 ± 5.7% for MWCNTs^18^. Interestingly, in presence of the antioxidant inducer *N*-acetyl-l-cysteine (NAC), the damages on both CNTs were significantly reduced, confirming the implication of oxidative stress in CNT degradation. In order to better quantify the kinetics of hole formation, the degradation rate was defined as the total surface degraded per time unit. No significant differences in the degradation speeds were observed between CNTs except for the period between 24 to 48 h. In this period, 0.9% of total surface area of MWCNTs were degraded each hour by macrophages compared with only 0.3%/h for Fe@MWCNTs (Fig. 1B). Interestingly, both CNTs were degraded at the same speed (0.16 vs 0.18%/h) between 48 and 168 h. NAC also significantly reduced the degradation speed of MWCNTs from 0.9%/h to 0.3%/h, but slightly reduced the degradation speed of Fe@MWCNTs from 0.3%/h to 0.2%/h (Fig. 1B) between 24 and 48 h. These results suggest either a difference in the production of ROS triggered by MWCNTs compared to Fe@MWCNTs or some role of iron oxide NPs in the degradation mechanism. To elucidate this point, ROS induced by MWCNTs or Fe@MWCNTs in the presence or absence of NAC were quantified by 2′,7′-dichlorodihydrofluorescein diacetate (DCFDA). ROS production was approximatively increased 3-fold regardless of the type of CNTs after 24 h and 48 h incubation and less than 1.5 times for both CNTs in the presence of NAC^18^. The absence of differences in ROS production supports the hypothesis of a transitory interference in the degradation mechanism due to the iron oxide NPs. In addition to the morphological transformation, Raman spectroscopy was used to characterize the chemical damages on CNTs as previously reported^14^,^19^,^27^. After 24, 48 and 168 h aging in cells and subsequent extraction, we observed for both CNTs an increased Raman intensity for the D-band (≈1,300 cm^−1^) in comparison to CNTs that were not exposed to cells but submitted to the same extraction protocol (control) (Fig. 1C and D). A marked loss of the G-band coupled with an increased D-band indicates that the graphene sidewalls were oxidized and degraded. The D/G ratio was larger for MWCNTs (0.33) than for Fe@MWCNTs (0.18) 48 h after the exposure, confirming slower chemical degradation of Fe@MWCNTs during the period 24–48 h after the exposure (Fig. 1E). Nevertheless, in agreement with morphological observations, the D/G ratio turned to be identical for both CNTs at 168 h, confirming the only transient effect of iron oxide NPs in the degradation mechanism. As iron is well-known to play a role in oxidative stress, effects of MWCNTs and Fe@MWCNTs on two iron-related-antioxidant-proteins, Ferritin H and Heme oxygenase 1, were investigated.

### Ferritin H and Heme oxygenase 1, two antioxidant proteins, are highly expressed in macrophages exposed to iron-free MWCNTs

**Figure 2:**
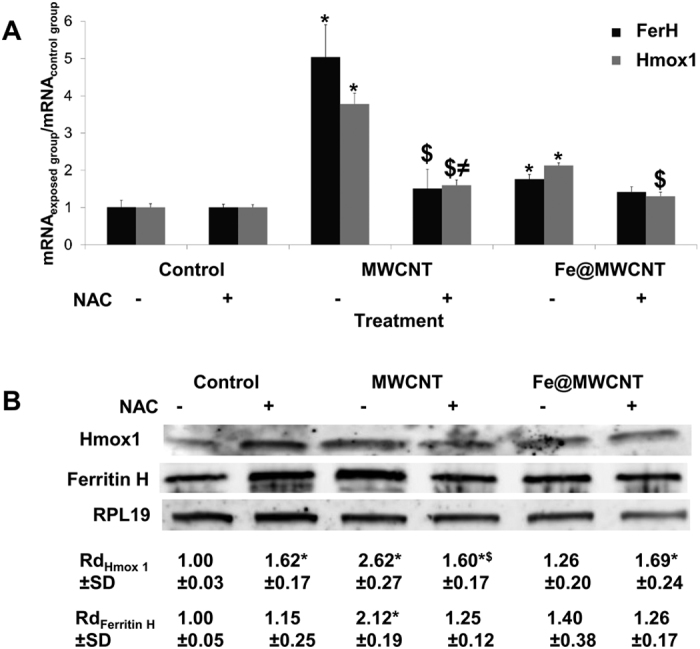
Effect of MWCNTs and Fe@MWCNTs on the gene expression and protein level of FerH and Hmox1 in the presence or absence of NAC. THP-1 differentiated into macrophages cells were treated with 1 μg/cm^2^ of MWCNT or Fe@MWCNT suspension in the presence or absence of NAC. After 24 h exposure, (**A**) mRNA quantification by qPCR or (**B**) protein level quantification by western blot analysis of FerH and Hmox1 were performed. Results are the mean ± SD of 3 separate experiments. (*) designates a statistically-significant difference from control group (p < 0.05). ($) designates a statistically-significant difference between MWCNT NAC or Fe@MWCNT NAC group and its respective equivalent MWCNT or Fe@MWCNT group without NAC treatment (p < 0.05). (≠) designates a statistically-significant difference between MWCNT NAC or Fe@MWCNT NAC and NAC control group (p < 0.05). Experiments were repeated at least three times with similar observations in each experiment. To improve the clarity, gels were cropped but full-length blots are presented in Supplementary Figure S1.

Heme degradation is critical for cellular defence through removal of prooxidant heme and increased production of bilirubin. Heme oxygenase 1 (Hmox1) catalyses the first step in the oxidative degradation of heme to form biliverdin and releases the heme iron in its ferrous form (Fe^2+^)^30^,^31^. The production of Fe^2+^ leads to the activation of iron regulatory protein (IRP) which is able to control the translation of iron sensitive protein like Ferritin H (FerH). In the absence of iron, IRP binds to ferritin mRNA and inhibits its translation. However, when iron ions are available, they bind to IRP and release IRP from ferritin mRNA, thus allowing its translation^32^. To understand the differential response of macrophages to MWCNTs and Fe@MWCNTs, we assessed the expression level of these two key proteins involved in iron-regulated oxidative stress mechanism. After 24 h exposure of macrophages to MWCNTs or Fe@MWCNTs, total mRNA were extracted to performed qPCR analyses. In the presence of MWCNTs, the mRNA levels of FerH and Hmox1 were increased by a factor of 5.04 and 3.78, respectively, in comparison to non-exposed cells. For Fe@MWCNTs, however, the mRNA levels of FerH and Hmox1 were increased by only 1.8 and 2.1, respectively (Fig. 2A). These results suggest an adaptive response of macrophages to CNTs depending on the presence or absence of iron oxide inside the nanotubes. In the presence of the antioxidant inducer, NAC, the over-expression of FerH and Hmox1 mRNA decreased to less than 1.5 times compared to control for both CNT types. This finding supports the important role of oxidative stress in the CNT-mediated induction of FerH and Hmox (Fig. 2A). Consistent results were obtained regarding the protein expression level quantified by western blot. FerH was induced 2.1 and 1.4 times after MWCNT and Fe@MWCNT exposure, respectively, while Hmox1 was induced 2.6 and 1.3 times (Fig. 2B). The protein expression levels were also strongly decreased in the presence of NAC (Fig. 2B), in line with qPCR results. The differences observed between mRNA and protein expression suggest a transcription and translational regulation of these genes after MWCNT and Fe@MWCNT exposure. In conclusion, both at the gene and protein levels, the antioxidant Hmox1 and FerH responses to CNT exposure are less marked in the presence of iron oxide.

### Nrf2 is more translocated to the nucleus of macrophages exposed to iron-free MWCNTs than to Fe@MWCNTs

**Figure 3:**
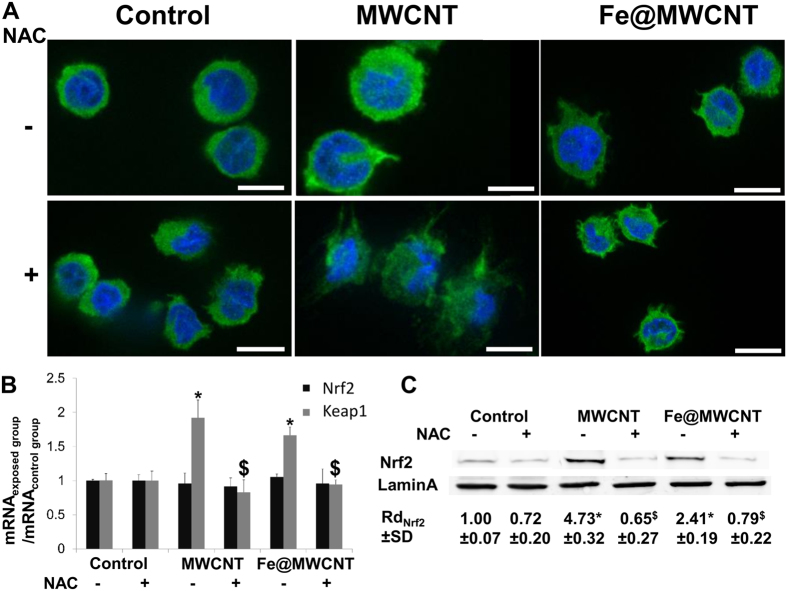
MWCNTs induce an oxidative stress-dependent nuclear translocation of Nrf2. THP-1 differentiated into macrophages cells were treated with 1 μg/cm^2^ of MWCNT or Fe@MWCNT suspension in presence or absence of NAC. After 24 h exposure, (**A**) immunofluorescence was performed to analyze Nrf2 nuclear translocation (scale bars correspond to 10 μm). (**B**) Nrf2 and Keap1 mRNA quantifications by qPCR were assessed to identify transcriptional regulation. (**C**) Western blotting on nuclear protein was performed to analyze Nrf2 nuclear translocation. Lamin A was used as nuclear marker. Experiments were repeated at least three times with similar observations in each experiment. Nrf2 was only translocated to the nucleus of cells exposed to MWCNTs. This phenomenon is inhibited by NAC suggesting the important role of oxidative stress in the nuclear translocation of Nrf2. To improve the clarity, gels were cropped but full-length blots are presented in Supplementary Figure S1.

Cellular defence against the toxicity of ROS comprises the enhancement of detoxifying enzymes, regulated by the antioxidant response element (ARE) that is located in the promoted region of related genes. Among the few transcription factor known to be activated by ROS, Nrf2 serves as a “master regulator” of cell survival through the coordinated induction of the phase II and antioxidant defence enzymes. Nrf2 has been shown to be associated with the Hmox1 response to multiple agents such as heme, cadmium, cobalt chloride, arsenite, nitroxyl heme, curcumin, caffeic acid, and carnosol as well as various electrophilic compounds and complex mixtures of phenolic compounds including diesel exhausts^33^. Activation of Nrf2 by oxidative stress is primarily controlled by the cytosolic inhibitor Kelch-like ECH-associated protein 1 (Keap1)^34^. Under basal conditions Keap1 retains Nrf2 in the cytoplasm and promotes its ubiquitination and degradation by the proteasome^34^. But, in the presence of oxidative stress, Keap1 is oxidized and Nrf2 nuclear translocation may occur^34^. Numerous prooxidant stimuli cause the dissociation of Nrf2 from Keap1 and permit the subsequent nuclear translocation of Nrf2 and its interaction with AREs of Hmox-1 promoter^34^. The pathway for promoting Hmox-1 thus depends of the activation of redox-dependent transcriptional activators such as Nrf2 along with the transcription repressor, BTB and CNC homolog 1 (Bach1). To determine the role of Nrf2 in the presence or absence of iron oxide embedded into CNTs, THP-1 differentiated into macrophages were exposed to MWCNTs and Fe@MWCNTs for 24 h at a concentration of 1 μg/cm^2^ and immunofluorescence assays using antibody against Nrf2 were performed. In the absence of CNTs, Nrf2 (green staining) was located almost exclusively in the cytoplasm, without or with very low level in the nucleus (blue DAPI staining) (Fig. 3A). Nrf2 was observed into the nucleus only after MWCNT exposure and to a lesser degree in the case of Fe@MWCNTs. Interestingly, NAC reduced Nrf2 nuclear translocation mediated by MWCNTs confirming the implication of oxidative stress in MWCNT-induced Nrf2 nuclear translocation. However, Nrf2 mRNA expression quantified by qPCR did not show any variations demonstrating an absence of transcriptional regulation but a modulation of Nrf2-nuclear translocation. In contrast, Keap1 mRNA was overexpressed 1.9 and 1.7 times in the presence of MWCNTs and Fe@MWCNTs, respectively, suggesting that Nrf2 nuclear translocation regulation is mediated by Keap1 (Fig. 3B). Western blot analyses of nuclear proteins extracted from cells exposed to MWCNTs and Fe@MWCNTs were in accordance with the immunofluorescence observations. Total Nrf2 was increased approximatively 5 times after MWCNT exposure and only 2.4 times after addition of Fe@MWCNTs in comparison to non-exposed cells (Fig. 3C).

### Bach1 is more translocated to the nucleus of macrophages exposed to Fe@MWCNTs than to iron-free MWCNTs

**Figure 4:**
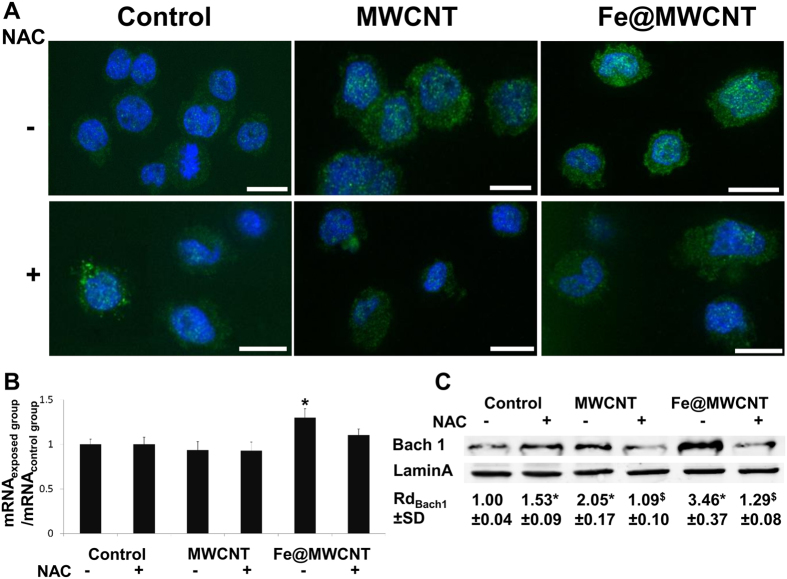
Fe@MWCNTs induce an oxidative stress dependent nuclear translocation of Bach1. THP-1 differentiated into macrophages cells were treated with 1 μg/cm^2^ of MWCNT or Fe@MWCNT suspension in presence or absence of NAC. After 24 h exposure, (**A**) immunofluorescence was performed to analyze Bach1 nuclear translocation (scale bars correspond to 10 μm). (**B**) Bach1 mRNA quantification by qPCR was assessed to identify transcriptional regulation. (**C**) Western blotting on nuclear protein was performed to analyze Bach1 nuclear translocation. Lamin A was used as nuclear marker. Experiments were repeated at least three times with similar observations in each experiment. Bach1 was only translocated to the nucleus of cells exposed to Fe@MWCNTs. This phenomenon is inhibited by NAC suggesting the important role of oxidative stress in the nuclear translocation of Bach1. To improve the clarity, gels were cropped but full-length blots are presented in Supplementary Figure S1.

Bach1 is also a crucial transcription factor involved in the oxidative stress response and it has a major role as a repressor of Hmox1 acting as an antagonist of Nrf2 activator^35^. To identify a potential role of Bach1 in CNT degradation mechanism, immunofluorescence assays using antibody against Bach1 were performed on THP-1 derived-macrophages exposed for 24 h to MWCNTs and Fe@MWCNTs at a concentration of 1 μg/cm^2^. Unlike the Nrf2 transcription factor, Bach1 (green) was preferentially located into the cytoplasm of non-exposed cells and translocated to the nucleus of cells exposed to Fe@MWCNTs and to a lesser extent to MWCNTs (Fig. 4A). As it was observed for Nrf2, NAC also inhibited CNT-mediated nuclear translocation of Bach1 (Fig. 4A). No or very low variations of mRNA were quantified by qPCR, suggesting a regulation at the protein level (Fig. 4B). Western blot analyses of nuclear proteins for Bach1 are in accordance with the immunofluorescence observations. Total Bach1 was translocated to the nucleus approximatively 2 times more after MWCNT exposure and 3.5 times more after Fe@MWCNT exposure (Fig. 4C) in comparison to non-exposed cells.

### Iron oxide nanoparticles embedded into Fe@MWCNTs are released and turned into free Fe^2+^ and Fe^3+^ in macrophages

**Figure 5:**
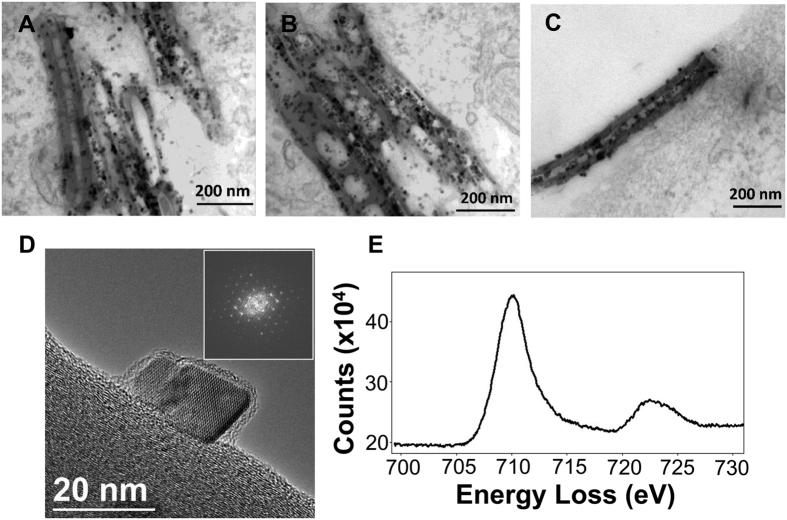
High-resolution analyses of Fe@MWCNTs. THP-1 differentiated into macrophages cells were treated with 1 μg/cm^2^ of Fe@MWCNT suspension for 48 h. (**A**,**B**) CNTs engulfed by macrophages or (**C**) at the surface of the cell were observed. (**D**) HRTEM image of an iron oxide nanoparticle released from the nanotube walls after 48 h aging in macrophages. The FFT of the HRTEM image allows identifying the inverse spinel structure of magnetite or maghemite. (**E**) EELS analysis of the Fe L_2,3_ edges was performed to distinguish between these two structures and confirmed the presence of magnetite. Experiments were repeated at least three times with similar observations.

To study the potential role of iron oxide into CNT degradation mechanism, we exposed macrophages to Fe@MWCNTs for 48 h and cells were stained for TEM observations. As we previously reported^18^, iron oxide NPs embedded into Fe@MWCNTs could be released from the tube structure engulfed into cells after macrophage-induced structural damages and creation of holes in the graphitic structure (Fig. 5A and B) but not on tubes observed at the cell surface (Fig. 5C). In other works, we also reported that iron oxide NPs of various shapes, including cubic shape, could be locally degraded and dissolved into the lysosomes of macrophages, thus releasing ionic iron species^36^,^37^. Depending of the initial structure of iron oxide, both Fe^2+^ and Fe^3+^ can be released from nanoparticles under acidic conditions like phagosome environment (pH≈4–5)^38^,^39^,^40^. To elucidate if iron species released from Fe@MWCNTs could have a role in their oxidative degradation, we identified the crystalline nature of the NPs released from Fe@MWCNTs after 48 h aging in the macrophages, using high resolution transmission electron microscopy (HRTEM) and electron energy loss spectroscopy (EELS). As shown in Fig. 5D, the observed inverse spinel structure could be associated to magnetite (made of both Fe^2+^ and Fe^3+^) or maghemite (with only trivalent cations). But the iron L_2,3_ edges obtained by EELS analyses is constituted by the two characteristic peaks of magnetite at 711 (L_3_) and 723 eV (L_2_) (Fig. 5E). The presence of maghemite in our experiment could be ruled out by the absence of a pre-peak at 709 eV on the L_3_ edge^41^. It is worth noticing that the 0.4 eV energy resolution of the microscope allows for detecting this peak splitting in iron oxides^42^. Hence, we unambiguously identified magnetite nanoparticles, which could release both Fe^2+^ and Fe^3+^ ions in acidic medium of phagosomes and participate to Haber-Weiss reaction and production of OH^•^.

## Discussion

**Figure 6:**
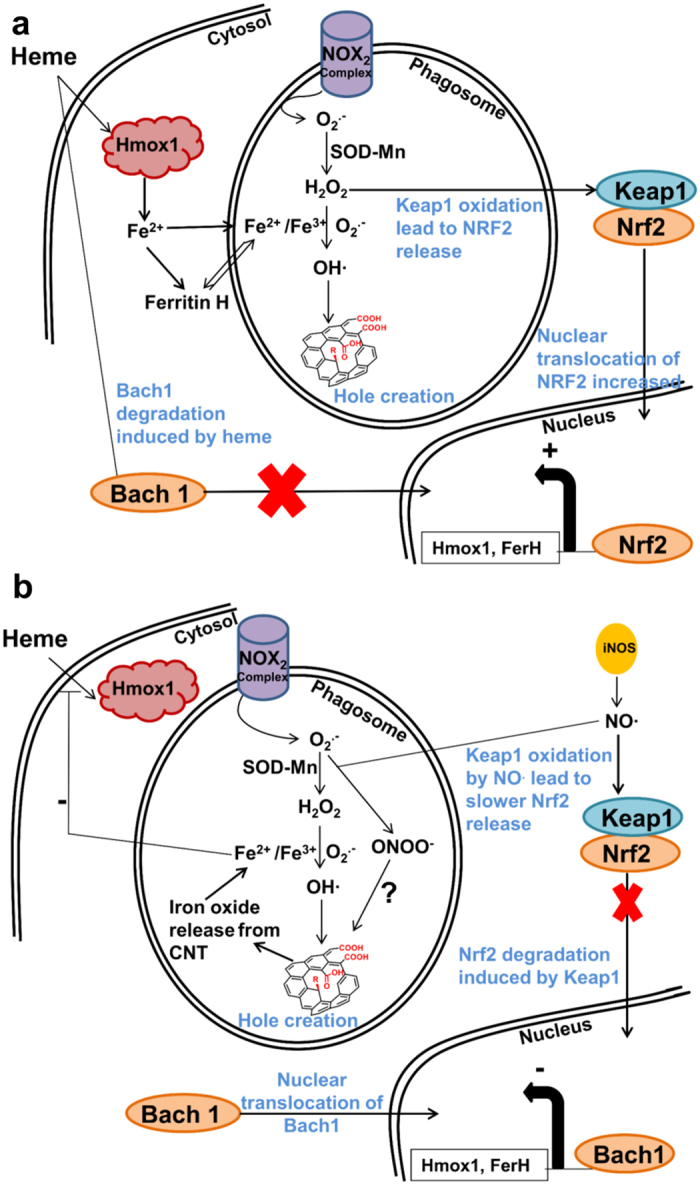
Schematic representation of the role of iron in MWCNT degradation mechanism in macrophages . After phagocytosis of CNTs, NOX_2_ complex is activated both on cytosolic and phagosomal membranes. Active NOX_2_ complex induced O_2_^•−^ production. O_2_^•−^ is turned into H_2_O_2_ by SOD into phagosome, and H_2_O_2_ is turned into OH^•^ in the presence of Fe^3+^ through the Haber-Weiss reaction. OH^•^ radicals then attack CNTs to generate carboxylic acids that create holes in the graphitic structure as we previously described^18^. (**a**) In the absence of iron embedded into the CNT structure, iron used for Haber-Weiss reaction has to be produced by the cells. Thus, oxidative stress products like H_2_O_2_ induce Keap1 cysteine oxidation and free Nrf2 for nuclear translocation. FerH and Hmox1 proteins, induced by Nrf2, will be translated for iron production. (**b**) In the presence of iron filled CNTs, iron from xenobiotics are converted into Fe^2+^ and Fe^3+^ in the acidic environment of the phagosome. Excess of iron ions inhibit heme entry in the cells and so induce Bach1 nuclear translocation and FerH and Hmox1 repression. As iNOS was induced only after Fe@MWCNT exposure, it is likely that Keap1 cysteine will be oxidized by NO^•^.

CNT degradation mechanism was previously reported to be associated with peroxynitrite pathway in macrophages^27^. Recently, we also reported another way adopted by macrophages to induce intracellular degradation of MWCNTs^18^. The mechanism described was based on the production of OH^•^ that induced nanotube damages, reduction of wall thickness and creation of holes in the graphitic structure. However, the production of OH^•^ in the phagosome of macrophages requires the presence of ferrous and ferric iron to initiate Haber-Weiss reaction. Thus, in the present study, we hypothesized a potential implication of iron-oxide embedded into CNTs or present as metal residue from CNT production in such intracellular degradation mechanism. After 48 h aging in the cells, Raman spectroscopy revealed a higher D-band/G-band ratio for MWCNTs than for iron-oxide loaded Fe@MWCNTs. Consistently, 48 h after exposure, the graphitic surfaces characterized by HRTEM were 1.7 times more degraded for MWCNTs than for Fe@MWCNTs, corresponding to a speed of degradation 2.7 times faster without iron. Quantification of ROS induced by both MWCNTs and Fe@MWCNTs revealed no differences 24 and 48 h after exposure. Taken together, these results suggest a slower CNT degradation in the presence of iron between 24 and 48 h. Moreover, when oxidative stress was limited by NAC, the degradation of MWCNTs was more inhibited than that of Fe@MWCNTs. Thus, the iron production needed for Haber-Weiss reaction was not regulated in the same way when iron comes from a cellular source or when iron is available in phagolysosome due to nanoparticle release from CNTs and potential dissolution. However, an initiation of CNT perforation must take place to start releasing iron embedded in CNTs. Moreover, the dissolution of iron oxide is a dynamic process that depends on pH and on the accessibility of iron chelators to the iron oxide surface^37^,^40^. At early time points, the low level of CNT holes does not allow to release CNT-embedded iron. To elucidate the delay observed in the response of macrophages to Fe@MWCNT exposure, we quantified the expression of two proteins involved in the iron metabolism associated to oxidative stress: Hmox1 and FerH. Hmox1 is well-known to catalyze heme proteins and release of Fe^2+^, whereas FerH, with its ferroxidase activity, is able to convert Fe^2+^ into Fe^3+^. Interestingly, both proteins were overexpressed after macrophage exposure to CNTs, but to a minor extent for Fe@MWCNTs in comparison to MWCNTs. FerH and Hmox1 expressions were described in previous studies about others xenobiotics^33^, to be regulated by Nrf2 and Bach1 transcription factors. Genes coding for these two proteins have an ARE sequence on their promotors which could be used by Nrf2 and Bach1 to induce and to repress their expressions, respectively. While no modification of Nrf2 and Bach1 mRNA was observed after both CNT exposure, we observed some differences at the protein level, with a larger nuclear translocation of Nrf2 and conversely a lower nuclear translocation of Bach1 for MWCNTs in comparison to Fe@MWCNTs. When translocated, Nrf2 probably induces FerH and Hmox1 translation via their ARE sequences, whereas Bach1 repressed FerH and Hmox1 translation due to its Nrf2 antagonist activity. Moreover, inhibiting the oxidative stress using NAC as antioxidant was shown to reduce MWCNT and Fe@MWCNT degradation. In the present study, we observed less Nrf2 and Bach1 translocation to the nucleus of cells pre-treated with NAC confirming the important role of oxidative stress in the activation of these transcription factors and in the expression of FerH and Hmox1 in response to CNTs. *In vivo,* in macrophages, Nrf2 activation is associated with the oxidation of Keap1 cysteines by H_2_O_2_ and/or O_2_^•−^43. Another mechanism based on the production of NO^•^ by iNOS to induce Keap1 oxidation and Nrf2 release in macrophages was also described^44^. However, Keap1 oxidation by NO^•^ is slower than oxidation by ROS leading to more Nrf2 degradation and, as a consequence, to less nuclear translocation. Previous quantification of iNOS mRNA level has shown an increase of 2.1 times after Fe@MWCNT exposure and no significant variation after MWCNT treatment^18^. In the light of the present study, we could hypothesize that iNOS acts as a regulator of Nrf2 release when intracellular iron level is too high. Our results are in accordance with a previous study performed on macrophages exposed to CNTs for 4 h which also revealed Nrf2 translocation and the implication of oxidative stress in this mechanism^45^. Although this study explored CNTs with different contents of iron residues (from 1.3 to 5.1% weight/weight), others parameters such as CNT diameter and length were not the same making difficult to understand the specific role of iron. Moreover, the early time-point used in that study (4 h) was likely too short to observe an effect of embedded iron on Nrf2 translocation and CNT degradation. On the other hand, several studies have reported an association between Hmox1 activity and FerH expression^46^,^47^,^48^. This phenomenon was related to a transient increase in iron caused by Hmox1-mediated heme degradation, which consequently relieved the post-translational repression of FerH. In our study, FerH mRNA is higher expressed than proteins (5.0 versus 2.1, respectively) after MWCNT treatment suggesting a regulation at the protein level. This difference between mRNA and protein level is less significant after Fe@MWCNT exposure (1.8 versus 1.4). In fact, the cells that have an excess of intracellular iron were able to modulate their Hmox1 and FerH transcription to reduce intracellular iron levels and maintain their iron homeostasis^32^. In our work, the lower level of Hmox1 and FerH after Fe@MWCNT exposure could be related to an increase of intracellular iron released from iron oxide-filled MWCNTs. This hypothesis is supported by the structure of iron oxide from Fe@MWCNTs, which was identified as magnetite. In the acidic conditions of phagosomes, magnetite can be partially dissolved and release Fe^2+^ and Fe^3+ ^39,40,49. Thus, once released, iron ions could inhibit intracellular heme uptake to maintain cell iron homeostasis. Interestingly, Bach1 binds with high affinity to heme, which inhibits its translocation and so its DNA-binding activity^50^. Thus, iron-inhibited heme uptake also promotes Bach1 nuclear translocation^35^,^51^, and so Hmox1 and FerH repression as it was observed after Fe@MWCNT exposure. All together these results allow us to propose a potential mechanism of CNT degradation in macrophages (Fig. 6). After engulfment of CNTs, NOX_2_ complex is activated on cytosolic and phagosomal membranes. Active NOX_2_ complex induced O_2_^•−^ production. Into phagosome, O_2_^•−^ is turned into H_2_O_2_ by superoxide dismutase (SOD) and H_2_O_2_ is transformed into OH^•^ in the presence of Fe^2+^ and Fe^3+^ (Haber-Weiss reaction). OH^•^ could attack CNTs to generate carboxylic acids creating holes in the graphitic structure as we previously reported^18^. In the absence of iron filled into CNT inner cavity, iron used for Haber-Weiss reaction should be produced by the cells. Thus, oxidative stress products like H_2_O_2_ are well-known to induce Keap1 cysteine oxidation freeing Nrf2 for nuclear translocation. FerH and Hmox1 proteins which could be induced by Nrf2 will be translated for iron production. Nevertheless, in the presence of iron filled CNTs, the released iron oxide nanoparticles will be converted into Fe^2+^ and Fe^3+^ due to the acidic environment of the phagosome. Excess of iron ions will inhibit heme entry in the cells and so favor Bach1 nuclear translocation and FerH and Hmox1 repression leading to a delay in the degradation mechanism. As described for other xenobiotics and given the induction of iNOS only after Fe@MWCNT exposure we hypothesized that Keap1 cysteine will be oxidized by NO^•^ with a slower rate than H_2_O_2_.

In conclusion, the ROS-induced degradation mechanism deployed by macrophages to clear carbon nanotubes is dependent on the intracellular iron metabolism and on the Nrf2/Bach1 signaling pathways. The cells require free intracellular Fe^3+^ to initiate oxidative reaction in response to CNTs, but should also tightly control the labile iron species to limit toxicity. Here, we have shown that iron oxide embedded into CNTs could interfere with the CNT clearance mechanism as a source of intracellular iron species. Macrophages regulate their extracellular uptake of iron to take into account the intracellular iron reservoir coming from CNTs and to maintain their iron homeostasis. The consequence is a delay in the chemical and structural degradation of CNTs induced by cells. In light of the present study we propose a Nrf2–regulated degradation mechanism in the presence or absence of iron inside CNTs. This mechanistic understanding of the ROS-induced MWCNT degradation could be exploited to modulate the degradation of CNTs using iron chelators and shed light on the molecular pathways governing the intracellular fate of CNTs.

## Materials and Methods

### Synthesis and functionalization of MWCNTs and Fe@MWCNTs

The production and functionalization of MWCNTs and Fe@MWCNTs has been reported in a previous study^11^. Briefly, pristine MWCNTs provided by Pyrograph Products (Cedarville, OH) with inner diameter from 40 to 80 nm were first washed with HNO_3_ to remove traces of the residual iron growth catalyst. The maximum number of oxygenated species on their surface was removed by heating at 900 °C under an inert atmosphere for 4 h. To produce Fe@MWCNTs, MWCNTs were dispersed in octadecene under ultrasonication, then iron stearate was added, and the solution was heated at 120 °C for 12 h to favor the diffusion of metal precursors inside MWCNTs. After another heating step (318 °C for 2 h), washing, centrifugation and filtration, the procedure for iron filling was once repeated. MWCNTs and Fe@MWCNTs were finally functionalized by arylation to introduce amino groups on their sidewall and increase their dispersibility in water^11^. Iron mass fraction was quantified by inductively coupled plasma atomic emission spectrometry revealing the absence of iron for MWCNTs and an iron mass fraction of 6.64% for Fe@MWCNTs^11^.

### Preparation and characterization of MWCNT and Fe@MWCNT suspensions

CNTs were suspended in cell culture media at a concentration of 100 μg/ml. The suspensions were sonicated for 5 min (5 s pause every 30 s) at 60 W with an ultrasonic bath (sonorex digitec, Bandelin). The quality of the suspensions was visually estimated and the agglomerates size was quantified using nanoparticle tracking analysis system (Nanosight, Malvern). Agglomerate mean size was quantified to be 114 ± 1.6 nm and 123.7 ± 1.5 nm for MWCNTs and Fe@MWCNTs respectively. Ninety percent of total agglomerates were smaller than 173 ± 2.8 nm and 194 ± 12.7 nm for MWCNTs and Fe@MWCNTs, respectively.

### Cellular tests

The human monocytic cell line THP-1 was obtained from ATCC (France) and cultured in fresh supplemented media (RPMI 1640 medium supplemented with 10% foetal calf serum (Gibco, France), 2 mM glutamine (Gibco, France), 100 U/ml penicillin, and 100 mg/ml streptomycin (Gibco, France)) at 37 °C and 5% CO_2_. The THP-1 cells were maintained between 10^5^ and 10^6^ cells/ml in fresh supplemented RPMI media. In the exponential phase of growth, cells were seeded onto 6-well plates at 2 × 10^6^ cells per well. After 48 h phorbol myristate acetate (PMA) treatment at 5 ng/ml, the cells were washed and exposed for 24 h to 1 μg/cm^2^ of MWCNTs or Fe@MWCNTs in RPMI media. All the suspensions were prepared extemporary in test tubes.

### MWCNT degradation in macrophages observed by Raman spectroscopy

Cells were seeded onto 75 cm^2^ flasks at 12 × 10^6^ cells per flask in presence of 5 ng/ml of PMA. After 48 h, cells were washed and fresh supplemented media was added for 24 h. Then, cells were treated with 1 μg/cm^2^ of MWCNTs or Fe@MWCNTs. After 24 h incubation time, cells were washed with PBS and fresh supplemented media was added in order to remove the non-phagocytized nanomaterials. At 168 h after exposure, cells were scraped and a centrifugation step at 350 g for 10 min was performed to separate the cells from the media. Cells were suspended in the presence of 200 μL of distilled water in a sonication bath at maximum power for 1 min, to lyse them by osmotic shock. Cell lysate was then centrifuged at 10000 g for 5 min. Supernatant was eliminated by pipetting and the pellet containing CNTs and cell residues was dispersed in water. After a final heating step at 80 °C for 15 min to remove the last cell residues, Raman spectroscopy was performed in solution using a Raman plus spectrometer (BW Tech) with a laser wavelength of 785 nm. Spectra were obtained over the range 1000 to 2000 cm^−1^ to visualize D- and G-bands at approximatively 1300 cm^−1^ and 1600 cm^−1^, respectively. Spectra were collected with a 30 s exposure time, at 100% laser power, and averaged across three scans per sample.

### High resolution transmission electron microscopy and electron energy loss spectroscopy

HRTEM imaging and EELS analyses were realized on a JEOL ARM 200 F microscope equipped together with a CEOS aberration corrector for the objective lens, a cold FEG and a GIF Quantum spectrometer. All the experiments were performed with a 80 kV acceleration voltage and an energy resolution of 0.4 eV^42^. Ten μL of the suspensions prepared for Raman spectroscopy assay were deposited on a carbon TEM grid and then dried for 5 min before TEM and HRTEM observations. To confirm that this extraction procedure did not affect the structure of the CNTs, a control sample was realized on 3 mL of suspension used to expose the cells. To compare CNTs extracted from cells and CNTs from control, both heating (37 °C for 168 h; 80 °C for 15 min) and centrifugation steps (350 g for 5 min and 10000 g for 5 min) were performed on the suspension in absence of cells. TEM analysis of this control sample revealed that the structure of CNTs is not disturbed by the extraction procedure.

### mRNA and protein quantifications of FerH, Hmox1, Nrf2 and Bach1 by reverse transcription real time polymerase chain reaction (RT-qPCR)

Cells were seeded onto 6-well plates at 2 × 10^6^ cells per well in presence of 5 ng/ml of PMA. After 48 h, cells were washed and fresh supplemented media was added for 24 h. Then, cells were pre-treated or not for 30 min with 10 mM of NAC and exposed to 1 μg/cm^2^ of MWCNTs or Fe@MWCNTs in the presence or absence of NAC. After 24 h, cells were washed with PBS and total RNA was extracted from cells with NucleoSpin RNA II^®^ Kit (Macherey Nagel) according to manufacturer’s protocol. One μg of total RNA was reverse transcribed to cDNA with SuperScript^®^ Reverse Transcriptase (Invitrogen, Cat n°18064–014) according to the manufacture’s protocol using Random primer (Promega, Cat no. C1181) and RNasin (Promega, Cat no. N2511). The quantification of mRNA for RPL19 (housekeeping gene, Fwd 5′-TCATCAAAGATGGGCTGATCAT-3′; Rev 5′-CATCGAGCCCGGGAATG-3′); Ferritin H (Fwd 5′-TGGCTTGGCGGAATTTCTGT-3′; Rev 5′-GCCCGAGGCTTAGCTTTCAT-3′); Heme oxygenase 1 (Fwd 5′- GCTTTCTGGTGGCGACAGTT -3′; Rev 5′- GCCAGCATGCCTGCATTC -3′); Nrf2 1 (Fwd 5′- AGCCCAGCACATCCAGTCA -3′; Rev 5′- TGTGGGCAACCTGGGAGTAG -3′); Bach1 1 (Fwd 5′- ACCTTGCCCATATGCTTGTGT -3′; Rev 5′- TCCTTCGGTGTCCGTCTCA -3′); Keap1 (Fwd 5′- GCCAAGCAAGAGGAGTTCTTCA -3′; Rev 5′- GTCGTCCCGGCTGATGAG -3′) transcripts were performed using SYBR^®^ green PCR master Mix (Applied Biosystems, Cat no.4367659) according to the manufacturer’s protocol on 300 ng of cDNA using StepOne plus Mastercycler (Applied Biosystems). Each sample was run in duplicate.

Protein quantifications were performed by western blot analysis on cells exposed as described for mRNA quantification. Proteins were extracted with Nucler/Cytosol fractionation Kit (Biovision) according to manufacturer’s protocol. Presence of Hmox1, FerH and RPL19 were analyzed on 50 μg of cytosolic proteins and Nrf2, Bach1 and lamin A on 15 μg of nuclear proteins by SDS-PAGE (4–20% polyacrylamide, Miniprotean TGX, Biorad). Separated proteins were transferred onto nitrocellulose membranes. Blots were saturated 2 h in TBST (Tris-base, pH 7.4, Tween 20%)-5% BSA and washed in TBST (3 × 5 min). They were subsequently incubated for 16 h at 4 °C with a rabbit Nrf2, Bach1, Hmox1, Ferritin H or lamin A antibody (Santa Cruz) or a goat RPL19 antibody (Santa Cruz) diluted at 1:300 in TBST-BSA 3%. After washing in TBST (3 × 5 min), the membranes were reacted with a goat anti-rabbit-HRP or a donkey anti-goat antibody (Santa Cruz) diluted at 1:3300 in TBST-BSA 3%. Presence of proteins was visualized with a chemiluminescent system (ECL+ Western blot detection kit, Amersham). Densitometry of each condition was calculated using ImageJ software. To compare the effect of each treatment, a Rd ratio was calculated. Cytosolic proteins (FerH or Hmox1) densitometry were normalized by RPL19 densitometry of each sample and nuclear proteins (Nrf2 and Bach1) densitometry were normalized by lamin A densitometry of each sample.


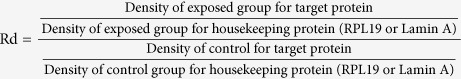


### Nuclear translocation of Nrf2 and Bach1 observed by immunofluorescence and confocal microscopy

4.5 × 10^5^ cells were grown in 4 chamber slides (LabTek) for 48 h in the presence of 5 ng/ml of PMA. After two wash steps, fresh supplemented media was added for 24 h, cells were pre-treated or not for 30 min with 10 mM of NAC and then exposed to 1 μg/cm^2^ of MWCNTs or Fe@MWCNTs in the presence or absence of NAC. Twenty-four hours after treatment, cells were washed and fixed with 4% formaldehyde for 15 min and permeabilized with 0.5% Triton X-100 in PBS for 10 min. Saturation of non-specific sites was performed with a solution containing 5% of BSA in PBS for 1 h. Rabbit anti-Nrf2 or anti-Bach1 (Santa Cruz) were diluted at 1/70 in a 3% PBS-BSA solution and incubated 1 h at 25 °C. FITC-conjugated goat anti-rabbit IgG (1:250) was used to label Nrf2 and Bach1 antibodies for 1 h at 25 °C. Cell nucleus were stained with a solution of UltraCruz^TM^ mounting medium (Santa Cruz) containing 1.5 μg/ml of DAPI. Confocal images were obtained by an Olympus JX81/BX61 Device/Yokogawa CSU Device spinning disk microscope (Andor Technology plc, Belfast, Northern Ireland).

### Analysis of iron oxide fate in macrophages

Cells were treated for 48 h by a dose of 1 μg/cm^2^ of Fe@MWCNT and fixed with 2.5% glutaraldehyde. After a postfixation with 1% osmium tetroxide, the samples were dehydrated by ethanol and embedded in EPON 812 (TAAB). The ultrathin sections of 90 nm for TEM analysis were obtained by an ultramicrotome (UCT, Leica), mounted on copper grids and stained with uranyl acetate and examined in on a JEOL ARM 200 F microscope equipped together with a CEOS aberration corrector for the objective lens, a cold FEG and a GIF Quantum spectrometer using an accelerating voltage of 80 kV.

### Analysis of iron oxide structure by HRTEM and EELS

HRTEM observations of iron oxide nanoparticles were performed in same conditions as described for CNTs. EELS spectra were recorded with a JEOL ARM 200 F microscope to cover a large energy range with an energy resolution of about 0.4 eV.

### Statistics

All data were expressed as mean ± S.D (standard deviation). F-test was used to compare the homogeneity of the variances. If homogeneity of variance was verified with a risk alpha equal to 5%, differences between each group were assessed with a one-way analysis of variance (ANOVA). When all ANOVA tests were positive, groups were subjected to the multiple-comparison Dunnett’s test. If the variances were not homogeneous, no significant differences between groups were considered. *P < 0.05 was considered as the statistical significance level.

## Additional Information

**How to cite this article**: Elgrabli, D. *et al*. Intracellular degradation of functionalized carbon nanotube/iron oxide hybrids is modulated by iron via Nrf2 pathway. *Sci. Rep.* **7**, 40997; doi: 10.1038/srep40997 (2017).

**Publisher's note:** Springer Nature remains neutral with regard to jurisdictional claims in published maps and institutional affiliations.

## Supplementary Material

Supplementary Information
