# Supplementary material for: Intracellular degradation of functionalized carbon nanotube/iron oxide hybrids is modulated by iron via Nrf2 pathway
Source: Sci Rep. 2017 Jan 25;7:40997. doi: 10.1038/srep40997 (PMC5264386; doi:10.1038/srep40997)
Supplement: Supplementary Information [file srep40997-s1.pdf]

Intracellular degradation of functionalized carbon nanotube/iron oxide hybrids is modulated by iron via Nrf2 pathway

Dan Elgrabli[1*], Walid Dachraoui[2], Hélène de Marmier[1], Cécilia Ménard-Moyon[3], Dominique Bégin[4], Sylvie Bégin[5], Alberto Bianco[3], Damien Alloyeau[2] and Florence Gazeau[1*]

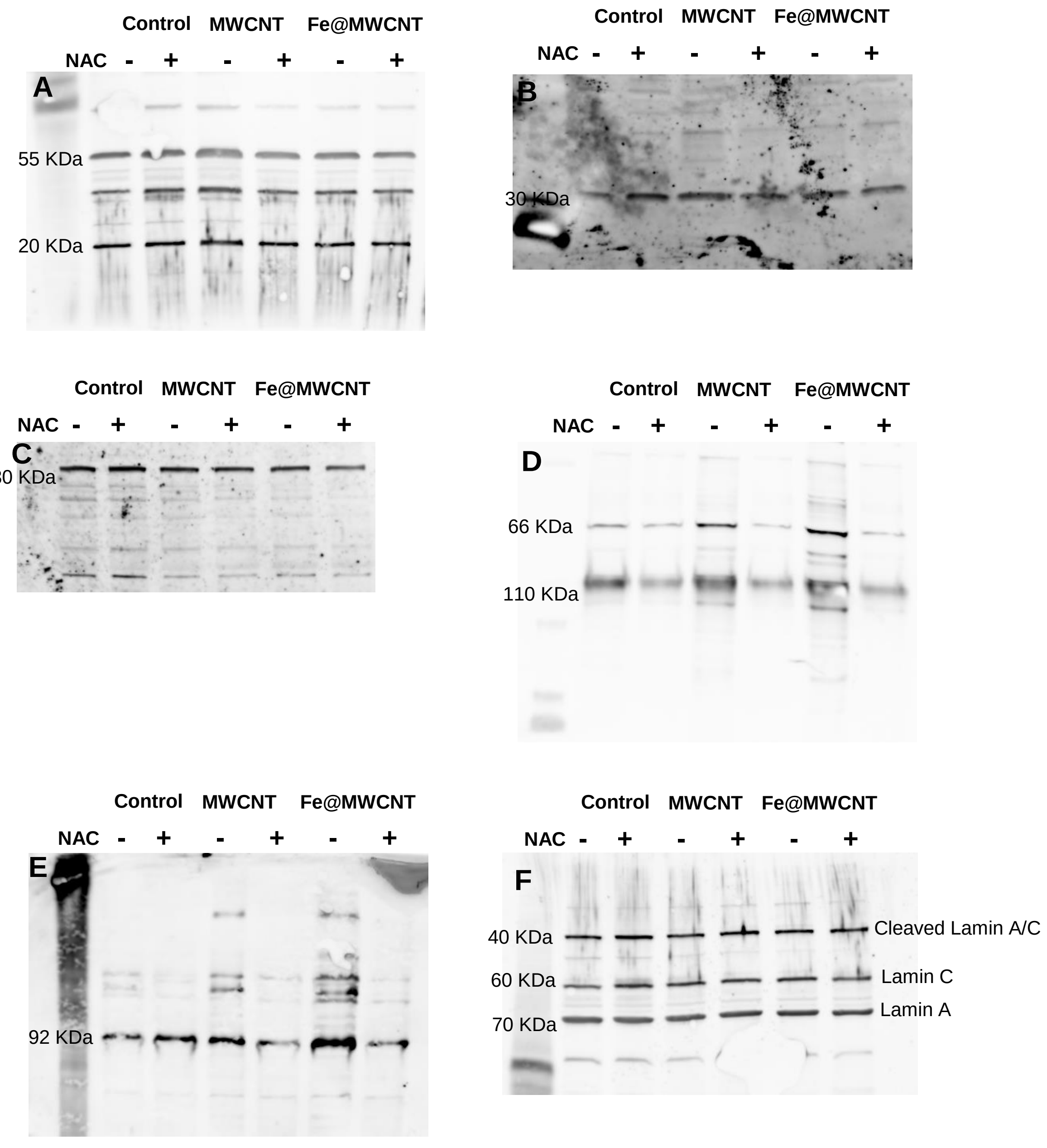

Fig S1: **Full western blots**. Ferritin H (A), Hmox1 (B), RPL19 (C), Nrf2 (D), Bach1 (E), Lamin A (F) full western blots corresponding to cropped blots displayed in fig 2 (Ferritin H, Hmox1 and RPL19), fig 3C (Nrf2 and lamin A) and fig 4C (Bach 1 and Lamin A). The commercial antibody from Santa Cruz Biotechnology for Nrf2 detect two bands at 66 and 110 kDa as observed in our experiment.
